# Supplementary material for: Analytical realization of complex thermal meta-devices
Source: Nat Commun. 2024 Jul 15;15:5527. doi: 10.1038/s41467-024-49630-1 (PMC11250795; doi:10.1038/s41467-024-49630-1)
Supplement: Supplementary file 1 — Supplementary Information [file 41467_2024_49630_MOESM1_ESM.pdf]

Supplementary Information: Analytical realization of complex thermal meta-devices

Weichen Li[1], Ole Sigmund[2], and Xiaojia Shelly Zhang[1,3]

[1]Department of Civil and Environmental Engineering, University of Illinois Urbana-Champaign, 205 North Mathews Ave, Urbana, 61801, IL, USA
[2]Department of Civil and Mechanical Engineering, Technical University of Denmark, Koppels Allé, Building 404, 2800 Kongens Lyngby, Denmark
[3]Department of Mechanical Science and Engineering, University of Illinois Urbana-Champaign, 1206 W. Green St, Urbana, 61801, IL, USA
*Corresponding author: zhangxs@illinois.edu

June 10, 2024

1 Supplementary Note 1: Transformation thermotics for thermal cloak, rotator, and concentrator

Transformations in thermotics as well as electromagnetic and other wave propagation problems are typically based on analytically derived coordinate transformation. However, transformation for non-trivial geometries may alternatively be obtained as solutions of non-linear, incompressible elastic problems as suggested in [1]. Here, however, we stick to the known analytical solutions from the literature based on transformation thermotics, involving cloaks, rotators, and concentrators.

With isotropic and homogeneous $\boldsymbol{\kappa}_0 = \kappa_0 \boldsymbol{I}$, the conductivity in the physical coordinate $\boldsymbol{\kappa} = \frac{\kappa_0}{\det \boldsymbol{J}} \boldsymbol{J}\boldsymbol{J}^{\mathrm{T}}$, where $\boldsymbol{J} := \frac{\partial \boldsymbol{x}}{\partial \bar{\boldsymbol{x}}} = \frac{\partial \boldsymbol{x}}{\partial \boldsymbol{s}} \frac{\partial \boldsymbol{s}}{\partial \bar{\boldsymbol{s}}} \frac{\partial \bar{\boldsymbol{s}}}{\partial \bar{\boldsymbol{x}}}$ is the Jacobian matrix corresponding to the coordinate transformation, and $\boldsymbol{s} := [r, \theta]$ and $\bar{\boldsymbol{s}} := [\bar{r}, \bar{\theta}]$ are the polar coordiantes in the physical and reference coordinates, respectively. Different thermal functions are realized by different $\bar{\boldsymbol{s}}$-$\boldsymbol{s}$ transformations.

For the cloak, the transformation is [2]:

$$r(\bar{r}, \bar{\theta}) = R_1(\bar{\theta}) + \frac{R_2(\bar{\theta}) - R_1(\bar{\theta})}{R_2(\bar{\theta})} \bar{r} \qquad \theta(\bar{\theta}) = \bar{\theta} \tag{1}$$

The resulting $\boldsymbol{\kappa}$ in the physical coordinate is:

$$\boldsymbol{\kappa} = \kappa_0 \boldsymbol{Q} \begin{bmatrix} \frac{r-R_1}{r}\left[1 + \left(\frac{R_1'}{r-R_1} + \frac{C'}{C}\right)^2\right] & \frac{R_1'}{r-R_1} + \frac{C'}{C} \\ \frac{R_1'}{r-R_1} + \frac{C'}{C} & \frac{r}{r-R_1} \end{bmatrix} \boldsymbol{Q}^{\mathrm{T}} \tag{2}$$

where $C(\bar{\theta}) := 1 - \frac{R_1(\bar{\theta})}{R_2(\bar{\theta})}$, $\boldsymbol{Q}$ is the standard rotation matrix characterized by θ, and the prime denotes derivative with respect to $\bar{\theta}$ (or θ equivalently).

For the rotator, the transformation is [2]:

$$r(\bar{r}) = \bar{r} \qquad \theta(\bar{r}, \bar{\theta}) = \bar{\theta} + \theta_0 \frac{f(R_2(\bar{\theta})) - f(\bar{r})}{f(R_2(\bar{\theta})) - f(R_1(\bar{\theta}))} \tag{3}$$

where θ_0 is the specified rotation angle, and $f(\cdot)$ is any smooth bijection function. Without loss of generality for engineering, we take $f(\bar{r}) = \bar{r}$. The resulting $\boldsymbol{\kappa} = \frac{\kappa_0}{\det \boldsymbol{T}} \boldsymbol{Q}\bar{\boldsymbol{\kappa}}\boldsymbol{Q}^{\mathrm{T}}$, where $\bar{\boldsymbol{\kappa}} = \boldsymbol{L}\boldsymbol{T}\boldsymbol{L}^{-2}\boldsymbol{T}^{\mathrm{T}}\boldsymbol{L}$ and $\boldsymbol{L} := \mathrm{diag}[1, r]$ and

$$\boldsymbol{T} := \begin{bmatrix} 1 & 0 \\ \theta_0 \frac{-1}{R_2 - R_1} & 1 + \theta_0 \frac{R_2'(R_2 - R_1) - (R_2 - r)\left(R_2' - R_1'\right)}{(R_2 - R_1)^2} \end{bmatrix} \tag{4}$$

Note that R_i and R'_i directly depend on the reference coordinate, and hence, given a physical coordinate pair (r, θ), we compute $R_i(\bar{\theta}(r, \theta))$ and $R'_i(\bar{\theta}(r, \theta))$. The inverse transformation $\bar{\theta}(r, \theta)$ for the rotator, unlike the cloak and concentrator, cannot be obtained in closed form in general and needs numerical solutions. We use bi-section to obtain $\bar{\theta}$. The solution exists and is unique as the mapping (coordinate transformation) is a bijection.

For the concentrator, an additional region enveloped by $R_3(\bar{\theta})$ between R_1 and R_2 is needed. The coordinate transformation is [2]:

$$\begin{aligned} r(\bar{r}, \bar{\theta}) &= \frac{R_1(\bar{\theta})}{R_3(\bar{\theta})}\bar{r} \qquad \theta(\bar{\theta}) = \bar{\theta} \qquad \text{if } \bar{r} \in [0, R_3] \\ r(\bar{r}, \bar{\theta}) &= \frac{R_1(\bar{\theta}) - R_3(\bar{\theta})}{R_2(\bar{\theta}) - R_3(\bar{\theta})}R_2(\bar{\theta}) + \frac{R_2(\bar{\theta}) - R_1(\bar{\theta})}{R_2(\bar{\theta}) - R_3(\bar{\theta})}\bar{r} \qquad \theta(\bar{\theta}) = \bar{\theta} \qquad \text{if } \bar{r} \in (R_3, R_2] \end{aligned} \tag{5}$$

Without loss of generality for engineering, we focus on the case $R_3(\bar{\theta}) = DR_1(\bar{\theta})$ with specified constant $D > 1$, i.e., R_3 is a dilation of R_1. This condition leads to isotropic $\boldsymbol{\kappa} = \kappa_0 \boldsymbol{I}$ in the core region. The resulting $\boldsymbol{\kappa}$ for $\bar{r} \in (R_3, R_2]$ (or $r \in (R_1, R_2]$) is $\boldsymbol{\kappa} = \frac{\kappa_0}{\det \boldsymbol{D}} \boldsymbol{Q}\bar{\boldsymbol{\kappa}}\boldsymbol{Q}^{\mathrm{T}}$, where $\bar{\boldsymbol{\kappa}} = \boldsymbol{L}\boldsymbol{D}\bar{\boldsymbol{L}}^{-2}\boldsymbol{D}^{\mathrm{T}}\boldsymbol{L}$ where $\bar{\boldsymbol{L}} := \operatorname{diag}[1, \bar{r}]$, $\boldsymbol{D}$ is given by

$$\boldsymbol{D} := \begin{bmatrix} \frac{R_2 - R_1}{R_2 - R_3} & \frac{\partial r}{\partial \bar{\theta}} \\ 0 & 1 \end{bmatrix} \tag{6}$$

and

$$\begin{aligned} \frac{\partial r}{\partial \bar{\theta}} =& R_2 \left[\frac{(R'_1 - R'_3)(R_2 - R_1) - (R_1 - R_3)(R'_2 - R'_3)}{(R_2 - R_3)^2} \right] + R'_2 \left(\frac{R_1 - R_3}{R_2 - R_3} \right) \\ &+ \bar{r} \left[\frac{(R'_2 - R'_1)(R_2 - R_3) - (R_2 - R_1)(R'_2 - R'_3)}{(R_2 - R_3)^2} \right] \end{aligned} \tag{7}$$

where the the prime denotes derivative with respect to $\bar{\theta}$ (or θ equivalently). Note the $\bar{r}$ in $\bar{\boldsymbol{L}}$ is the reference coordinate, which can be obtained through the inverse transformation with analytical expression given (r, θ).

2 Supplementary Note 2: Definition of meta-device domains

This section provides the definition of inner and outer boundaries used in this study in the form of $R_1(\bar{\theta})$ and $R_2(\bar{\theta})$, respectively. Both boundaries take the form

$$R(\bar{\theta}) = R_0 + \sum_{i=1}^{I} A_i \cos\left(T_i(\bar{\theta} + \psi_1)\right) + \sum_{j=1}^{J} B_j \sin\left(S_j(\bar{\theta} + \psi_2)\right) \tag{8}$$

where R_0, A_i, T_i, B_j, S_j, ψ_1 and ψ_2 are prescribed parameters. We provide the values of these parameters for the three thermal meta-devices below with unmentioned parameters equal to zero.

For the flower-shaped cloak, the inner boundary parameters are $R_0 = 13.2458$, $A_1 = 1.5583$, $T_1 = 1$, $B_1 = 0.7792$, $B_2 = -1.5583$, $S_1 = 2$, and $S_2 = 3$. The outer boundary parameters are $R_0 = 29.2188$, $A_1 = 3.8958$, $T_1 = 5$, $B_1 = 1.9479$, $B_2 = -1.9479$, $S_1 = 1$, and $S_2 = 2$.

For the shuriken-shaped rotator, the inner boundary parameters are $R_0 = 13.8$, $A_1 = 2.3$, $T_1 = 2$, and ψ_1 is the same as the prescribed rotated angles. The outer boundary parameters are $R_0 = 30$, $A_1 = 6$, $T_1 = 4$, and $\psi_2 = 0$.

For the heart-shaped concentrator, the inner boundary parameters are $R_0 = 13.8$, $A_1 = 2.3$, $T_1 = 3$, $B_1 = 0.5$, $B_2 = -1.1$, $S_1 = 2$, and $S_2 = 3$. The outer boundary parameters are $R_0 = 28.8$, $A_1 = 4.8$, $T_1 = 2$, $B_1 = 2.4$, $B_2 = 4.8$, $S_1 = 1$, and $S_2 = 3$.

3 Supplementary Note 3: Rank-2 laminate and its conversion to single-scale microstructure

The rank-2 laminates are made of two isotropic constituents (Materials A and B) with conductivity κ_A and κ_B, respectively. For convenience and without loss of generality, we assume $\kappa_A \geq \kappa_B$. The volume

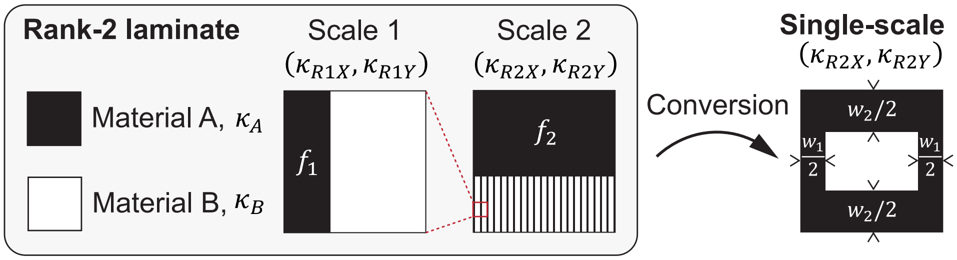

Supplementary Figure 1: Rank-2 laminate and its conversion to a single-scale structure

fractions of Material A at the two scales are denoted by $f_1 \in [0,1]$ and $f_2 \in [0,1]$, respectively. Based on this setup, we will first introduce the forward process of computing rank-2 laminate's homogenized conductivities given f_1 and f_2, then elaborate on the inverse process of how to obtain f_1 and f_2 given the target homogenized conductivity. The inverse process is needed to generate the microstructures of the thermal meta-devices.

3.1 Homogenized conductivity of rank-2 laminates

With the orientation in Supplementary Figure 1, the homogenized thermal conductivity of the rank-2 laminate in the two principal directions (X and Y) can be obtained through:

$$\begin{aligned} \kappa_{R2,X} &= f_2 \kappa_A + (1 - f_2)\, \kappa_{R1,X} \\ \kappa_{R2,Y} &= \frac{1}{\frac{f_2}{\kappa_A} + \frac{(1-f_2)}{\kappa_{R1,Y}}} \end{aligned} \tag{9}$$

where $\kappa_{R1,X}$ and $\kappa_{R1,Y}$ are the principal homogenized conductivities of the rank-1 laminate obtained by

$$\begin{aligned} \kappa_{R1,X} &= \frac{1}{\frac{f_1}{\kappa_A} + \frac{(1-f_1)}{\kappa_B}} \\ \kappa_{R1,Y} &= f_1 \kappa_A + (1 - f_1)\, \kappa_B \end{aligned} \tag{10}$$

Rank-2 laminates can achieve all physically permissible homogenized $(\kappa_{R2,X}, \kappa_{R2,Y})$ pairs. This is demonstrated in Supplementary Figure 2 which shows the complete range of achievable homogenized $(\kappa_{R2,X}, \kappa_{R2,Y})$ given $\kappa_A = 17.9 W m^{-1} K^{-1}$ (Steel) and $\kappa_B = 0.16 W m^{-1} K^{-1}$ (PDMS). The range is obtained by sweeping f_1 and f_2, and it is bounded by the rank-1 laminate's $\kappa_{R1,X} - \kappa_{R1,Y}$ functions when sweeping f_1. This also demonstrates the huge advantage of rank-2 laminate over rank-1 as the latter's achievable $(\kappa_{R1,X}, \kappa_{R1,Y})$ range is a degenerated subset (denoted by the thick borders in Supplementary Figure 2). We also show several rank-2 laminates corresponding to various homogenized conductivities in Supplementary Figure 2.

3.2 Inverse homogenization of rank-2 laminate

Realizing the thermal meta-device requires the inverse homogenization of rank-2 laminates, that is, given the two eigenvalues of $\boldsymbol{\kappa}$ obtained from transformation thermotics, what are the f_1 and f_2 values such that the corresponding homogenized $\kappa_{R2,X}$ and $\kappa_{R2,Y}$ from (9) are equal to the two eigenvalues? The inverse problem has a closed-form solution attained by solving (9) and (10) for f_1 and f_2 given $\kappa_{R2,X}$ and $\kappa_{R2,Y}$:

$$\begin{aligned} f_1 &= \frac{\kappa_A \left(\kappa_A \kappa_B - \kappa_A \kappa_{R2,X} - \kappa_B \kappa_{R2,X} + \kappa_{R2,X} \kappa_{R2,Y}\right)}{-\kappa_A^3 + \kappa_A^2 \kappa_B + \kappa_A \kappa_{R2,X} \kappa_{R2,Y} - \kappa_B \kappa_{R2,X} \kappa_{R2,Y}} \\ f_2 &= \frac{\kappa_A \left(\kappa_A \kappa_B - \kappa_A \kappa_{R2,Y} - \kappa_B \kappa_{R2,Y} + \kappa_{R2,X} \kappa_{R2,Y}\right)}{-\kappa_A^3 + \kappa_A^2 \kappa_{R2,X} + \kappa_A \kappa_B \kappa_{R2,X} - \kappa_B \kappa_{R2,X} \kappa_{R2,Y}} \end{aligned} \tag{11}$$

where physical values are $f_1, f_2 \in [0,1]$. In (11), $\kappa_{R2,X}$ and $\kappa_{R2,Y}$ are set equal to the two eigenvalues of $\boldsymbol{\kappa}$.

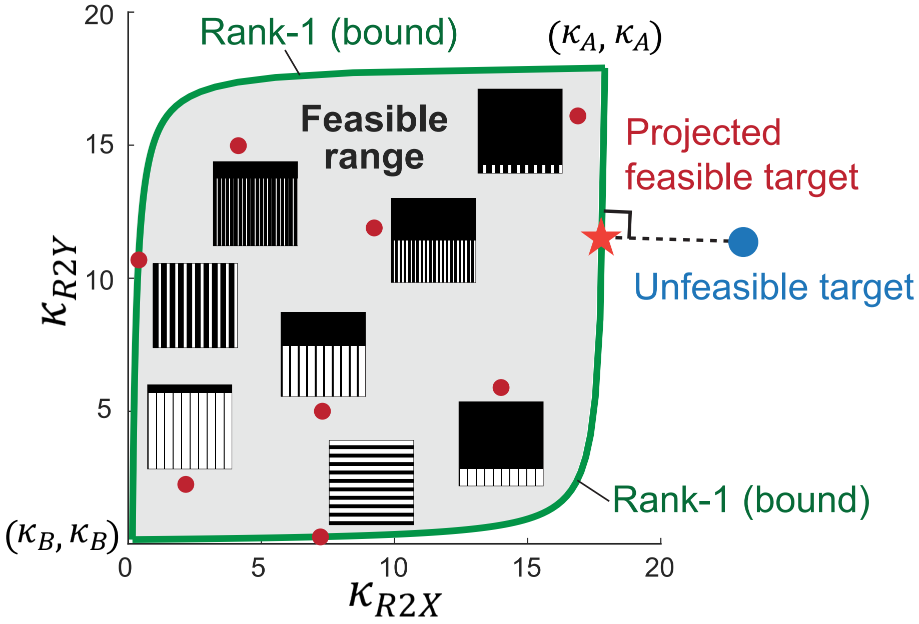

Supplementary Figure 2: Feasible range of rank-2 laminate's homogenized conductivity bounded by rank-1 laminate's homogenized conductivity with selected samples of rank-2 laminates ($\kappa_A = 17.9Wm^{-1}K^{-1}$ and $\kappa_B = 0.16Wm^{-1}K^{-1}$) and projection of infeasible target.

Importantly, given the real material properties of κ_A and κ_B, not arbitrary target $\kappa_{R2,X}$ and $\kappa_{R2,Y}$ values from transformation thermotics are achievable. To produce meaningful f_1 and f_2, the target values need to be within the feasible regions indicated in Supplementary Figure 2. For outlying targets, we perform a least-distance projection back to the feasible region and use the projected values as the target $\kappa_{R2,X}$ and $\kappa_{R2,Y}$ for computing f_1 and f_2 as indicated in Supplementary Figure 2. As the feasible region is convex, the projected target is unique and is obtained by the bi-section method. In most of the cases studied herein, the outlying targets from transformation thermotics are small in proportion, and most targets are within the feasible region. However, one may use the achievability of the target values as a means to look for appropriate material constituents for a given thermal manipulation task.

3.3 Volume-equivalent conversion of rank-2 laminate to single-scale microstructure

The rank-2 laminate is two-scale, but the de-homogenization method aims at a manufacturable single-scale setup and hence requires the conversion of the analytical rank-2 to a single-scale microstructure. Here, we use a volume-equivalent formula [3, 4] to analytically convert the rank-2 laminate defined by f_1 and f_2 to a single-scale microstructure defined by the thicknesses of Material A regions w_1 and w_2 in the two principal directions, as illustrated in Supplementary Figure 1. The conversion is carried out by computing the following quantities as functions of f_1 and f_2.

$$\begin{aligned} &\eta := 1 - (1 - f_1)(1 - f_2) \\ &D_1 := \frac{f_1}{\eta}; \qquad D_2 := \frac{(1 - f_1) f_2}{\eta} \\ &\Delta := D_1^2 + D_2^2 + 2(1 - 2\eta) D_1 D_2 \\ &\beta := \frac{(D_1 + D_2) - \sqrt{\Delta}}{2 D_1 D_2} \end{aligned} \tag{12}$$

Finally, the widths are given as $w_1 = \beta D_1$ and $w_2 = \beta D_2$. For the derivation and more details of the formulas, readers are referred to [3, 4].

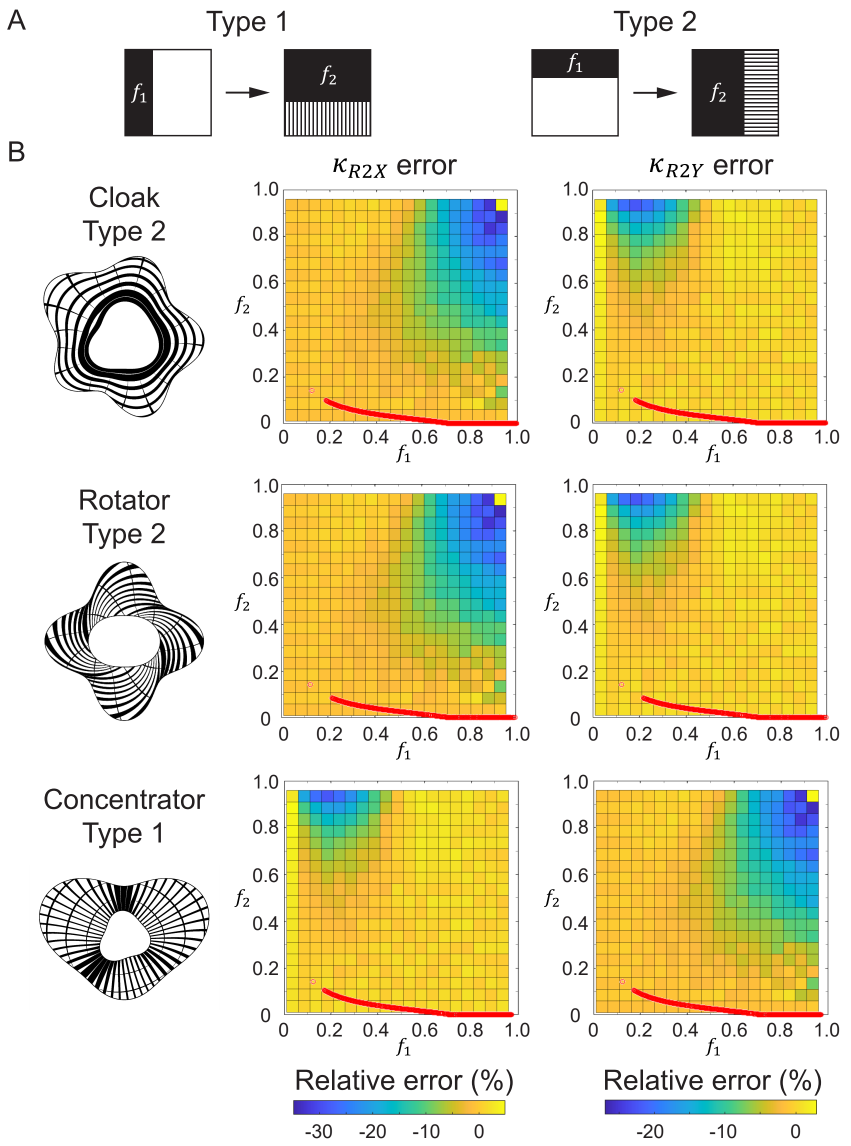

Supplementary Figure 3: Error from the single-scale conversion as a function of f_1 and f_2. A) Two types of lamination; B) error distribution and f_1-f_2 coordinates for the cloak, rotator, and concentrator.

The volume-equivalent conversion inevitably perturbs the homogenized conductivity, i.e., the homogenized conductivity of the single-scale microstructure obtained by (12), in general, differs from the corresponding rank-2 laminate's, but the differences are relatively small for all the studied cases. This is demonstrated in Supplementary Figure 3, which shows the relative errors between the two as a function of f_1 and f_2, where the homogenized conductivity of the single-scale microstructure is obtained by numerical homogenization. Specifically, the errors (take X-direction as an example) are defined as $(\kappa_{HX}(f_1, f_2) - \kappa_{R2X}(f_1, f_2))/\kappa_A$, where κ_{HX} is obtained by numerical homogenization of the single-scale converted microstructure defined by $w_1(f_1, f_2)$ and $w_2(f_1, f_2)$.

The error fringe plot in Supplementary Figure 3B shows that for most f_1-f_2 regions (shown in yellow), the errors are within 5%, but for regions with large f_1 and f_2, the errors are magnified. Importantly, for regions with large f_1-f_2 differences, which indicate high anisotropy, the errors are generally small. These regions happen to host the metamaterial structures' f_1-f_2 pairs as labeled by the red markers over the fringe plots. This demonstrates that the errors from the single-scale conversion are generally small for all the studied cases.

4 Supplementary Note 4: De-homogenization: closed-form solution of $\boldsymbol{\phi}$ for cloak and concentrator with circular disk domain

For cloak and concentrator with circular domains, $\boldsymbol{\kappa} = \kappa_0 \boldsymbol{Q}\bar{\boldsymbol{\kappa}}\boldsymbol{Q}^{\mathrm{T}}$ with $\bar{\boldsymbol{\kappa}} = \mathrm{diag}[\frac{r-R_1}{r}, \frac{r}{r-R_1}]$ for the cloak and $\bar{\boldsymbol{\kappa}} = \mathrm{diag}[\frac{r-d}{r}, \frac{r}{r-d}]$ with $d := R_2\frac{R_1-R_3}{R_2-R_3}$ for the concentrator, respectively. As both $\bar{\boldsymbol{\kappa}}$ are diagonal, the rotation matrix $\boldsymbol{Q}$ is the eigenmatrix of $\boldsymbol{\kappa}$ for both meta-devices, with eigenvectors $\mathbf{v}_1 = [\cos\theta, \sin\theta]^{\mathrm{T}}$ and $\mathbf{v}_2 = [-\sin\theta, \cos\theta]^{\mathrm{T}}$ (the first and second column of $\boldsymbol{Q}$, respectively). We take the guiding vectors $\mathbf{e}_1 = \mathbf{v}_2$ and $\mathbf{e}_2 = \mathbf{v}_1$. Importantly, the angle field θ is harmonic (Laplacian-free), which is necessary for the existence of a conformal mapping [5].

Based on the above notation, the problem to find the conformal mapping $\boldsymbol{\phi} := [\phi_1, \phi_2]^{\mathrm{T}}$ can be stated as: find scalar fields $p > 0$, ϕ_1, and ϕ_2 such that $\nabla\phi_1 = p\mathbf{e}_1$ and $\nabla\phi_2 = p\mathbf{e}_2$. For simply connected domains, this implies that $p\mathbf{e}_1$ and $p\mathbf{e}_2$ are curl-free (gradient fields), i.e., $\nabla \times (p\mathbf{e}_1) = \mathbf{0}$ and $\nabla \times (p\mathbf{e}_2) = \mathbf{0}$. This requirement yields the solution for p, with which we can integrate to get ϕ_1 and ϕ_2.

Radial symmetry of the disk domain implies that p is a function of r only. With this simplification, $p\mathbf{e}_2$ is identically curl-free. For $p\mathbf{e}_1$, imposing the curl-free condition leads to the following ODE of p:

$$p' + \frac{p}{r} = 0 \tag{13}$$

The solution is $p(r) = \frac{C_0}{r}$ with constant $C_0 > 0$. Plugging back the solution of p to $\nabla\phi_1 = p\mathbf{e}_1$ and $\nabla\phi_1 = p\mathbf{e}_2$ yields:

$$\begin{aligned} \phi_1(\theta) &= C_0\theta + C_1 \\ \phi_2(r) &= C_0 \ln r + C_2 \end{aligned} \tag{14}$$

where C_1 and C_2 are integration constants.

Importantly, the disk domain without modification is not simply connected as assumed in the derivation. To ensure the domain is simply connected, one can restrict the domain to the open set $\{(r,\theta)|\ r \in [R1, R2], \theta \in (0, 2\pi)\}$,i.e., the disk domain with X-axis removed. Indeed, this is required to ensure the solution of ϕ_1 is a well-defined function.

5 Supplementary Note 5: De-homogenization: Numerical least-square solution for arbitrary domain

A conformal mapping $\boldsymbol{\phi}$ may not exist for non-circular domains because the angle field of the eigenvectors (or guiding vectors) of $\boldsymbol{\kappa}$ is, in general, non-harmonic. Hence, we adopt a numerical least-square approach with the Finite Element Method (FEM) to obtain the approximate solution of ϕ_1 and ϕ_2. Here, we present the procedure of numerically solving for ϕ_1 (the procedure for ϕ_2 is the same). We

use the penalty method to solve the equality-constrained least-sqaure problem (Equation (1) of the main text). The Lagrangian is

$$\min_{\phi_1 \in S} L[\phi_1] := \int_\Omega ||\nabla\phi_1 - \mathbf{e}_1||^2 \, \mathrm{d}\Omega + \lambda \int_\Omega \frac{(\nabla\phi_1 \cdot \mathbf{e}_2)^2}{\nabla\phi_1 \cdot \nabla\phi_1} \, \mathrm{d}\Omega \tag{15}$$

where S is the set with proper boundary conditions, λ is the penalty parameter. Note that the penalty term in (15) is normalized with $\nabla\phi_1 \cdot \nabla\phi_1$ to penalize only the angle error (and not the length error) between $\nabla\phi_1$ and $\mathbf{e}_2$.

5.1 Finite element discretization

The least-square problem (15) is solved using FEM. We use the four-node quadrilateral bilinear element and adopt a piece-wise constant (within each element) distribution of $\mathbf{e}_1$ and $\mathbf{e}_2$ evaluated at the element centroids. We use a second-order Gaussian quadrature with four Gauss points. The FE-discretized form of (15) is:

$$\min_{\tilde{\boldsymbol{\phi}}_1} \tilde{L}(\tilde{\boldsymbol{\phi}}_1) := \sum_{e=1}^{N} \left\{ \int_{\Omega_e} ||\boldsymbol{B}^{(e)} \tilde{\boldsymbol{\phi}}_1^{(e)} - \mathbf{e}_2^{(e)}||^2 \, \mathrm{d}\Omega + \lambda \int_{\Omega_e} \frac{(\boldsymbol{B}^{(e)} \tilde{\boldsymbol{\phi}}_1^{(e)} \cdot \mathbf{e}_2^{(e)})^2}{\boldsymbol{B}^{(e)} \tilde{\boldsymbol{\phi}}_1^{(e)} \cdot \boldsymbol{B}^{(e)} \tilde{\boldsymbol{\phi}}_1^{(e)}} \, \mathrm{d}\Omega \right\} \tag{16}$$

where $\tilde{\boldsymbol{\phi}}_1$ is the vector of global nodal values, $\boldsymbol{B}^{(e)}$ is the matrix of shape function gradient of element e (a total of N elements). The value of $\tilde{\boldsymbol{\phi}}_1$ at one node is set to zero to ensure proper boundary conditions.

We use Newton's method to solve (16) with a continuation of λ from 1 to 10^4 by a factor of 2 outside the Newton iterations. With the notation of $\mathbf{F}_1 := \boldsymbol{B}^{(e)} \tilde{\boldsymbol{\phi}}_1^{(e)}$, $I_{11} := \mathbf{F}_1 \cdot \mathbf{e}_1$, $I_{12} := \mathbf{F}_1 \cdot \mathbf{e}_2$, and $J_1 := \mathbf{F}_1 \cdot \mathbf{F}_1$, and omitting the (e) for convenience, the element-level gradient and Hessian of $\tilde{L}$ are given by:

$$\boldsymbol{g}^{(e)}(\tilde{\boldsymbol{\phi}}_1^{(e)}) := \int_{\Omega_e} \boldsymbol{B}^{\mathrm{T}} \left(\frac{\partial J_1}{\partial \mathbf{F}_1} - 2\frac{\partial I_{11}}{\partial \mathbf{F}_1} \right) \mathrm{d}\Omega + \lambda \int_{\Omega_e} \boldsymbol{B}^{\mathrm{T}} \frac{1}{J_1^2} \left(2 I_{12} J_1 \frac{\partial I_{12}}{\partial \mathbf{F}_1} - I_{12}^2 \frac{\partial J_1}{\partial \mathbf{F}_1} \right) \mathrm{d}\Omega \tag{17}$$

$$\begin{aligned} \boldsymbol{h}^{(e)}(\tilde{\boldsymbol{\phi}}_1^{(e)}) := & 2\int_{\Omega_e} \boldsymbol{B}^{\mathrm{T}} \boldsymbol{B} \mathrm{d}\Omega + \lambda \int_{\Omega_e} \boldsymbol{B}^{\mathrm{T}} \left\{ -\frac{2}{J_1^3} \left(2 I_{12} J_1 \frac{\partial I_{12}}{\partial \mathbf{F}_1} - I_{12}^2 \frac{\partial J_1}{\partial \mathbf{F}_1} \right) \otimes \frac{\partial J_1}{\partial \mathbf{F}_1} \right. \\ & \left. + \frac{1}{J_1^2} \left(2 J_1 \frac{\partial I_{12}}{\partial \mathbf{F}_1} \otimes \frac{\partial I_{12}}{\partial \mathbf{F}_1} + 2 I_{12} \left(\frac{\partial I_{12}}{\partial \mathbf{F}_1} \otimes \frac{\partial J_1}{\partial \mathbf{F}_1} - \frac{\partial J_1}{\partial \mathbf{F}_1} \otimes \frac{\partial I_{12}}{\partial \mathbf{F}_1} \right) - I_{12}^2 \frac{\partial^2 J_1}{\partial \mathbf{F}_1^2} \right) \right\} \boldsymbol{B} \mathrm{d}\Omega \end{aligned} \tag{18}$$

where $\otimes$ denotes the outer product. The same procedure applies to solving ϕ_2 and is not repeated herein.

We use a pixel square mesh for solving the FE problem. We first generate a 400×400 mesh for a square domain containing the meta-device domains. Then, the elements inside the core (enveloped by R_1) and outside the meta-device (out of R_2) are removed. The remaining pixel mesh covers the meta-device domain bounded by R_1 and R_2. The value of a node is set to 0 to avoid singularity and non-uniqueness of the solution.

The FE problem is implemented and solved using Matlab R2020b [6] with a desktop workstation (AMD Ryzen Threadripper 3970X 32-Core Processor 3.69 GHz, 256 GB RAM). Parallel *for* loop is used for looping over the elements' gradient and Hessian. The total computational cost for solving the nonlinear FE problem for the global fields ϕ_1 and ϕ_2 is less than 30 seconds, which is many orders of magnitude more efficient than state-of-the-art numerical methods for generating local microstructures.

5.2 Special treatments for ensuring simple connectedness

Direct use of a FE domain enclosed by R_1 and R_2 will violate the simply connected condition, and hence, special treatment is required in the FE problem (16). To this end, we introduce a zero-width gap at the positive X-axis as illustrated in Supplementary Figure 4A by separating each FE node at the gap into two overlapped but unconnected nodes. The node separation ensures a simply connected domain, but it alone cannot ensure smooth connection at the gap as the members on the two sides can mismatch. The direct cause of the mismatch is the small difference in the distribution of $\nabla\phi_1$ (take ϕ_1

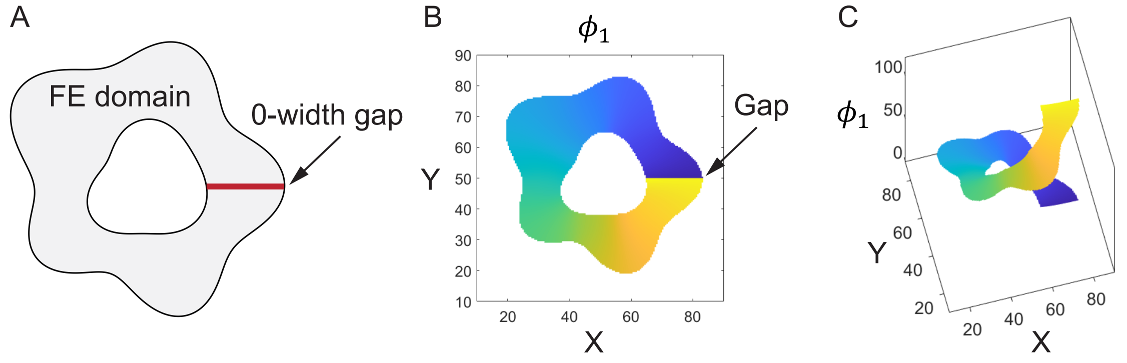

Supplementary Figure 4: Special treatments to ensure simply connected domains. A) FE domain with a 0-width gap; B) distribution of ϕ_1 of the cloak; C) graph of ϕ_1.

for illustration, same for ϕ_2) on the two sides of the gap. Such difference should be eliminated because the two sides of the gap are essentially the same location with the same $\boldsymbol{\kappa}$, $\mathbf{e}_1$, and $\mathbf{e}_2$. Their presence is mainly due to the least-square fit.

To have identical distributions of $\nabla\phi_1$ at the gap, we require ϕ_1 on the two sides to differ by only a constant scalar. We directly impose such a constraint on the DOFs of the two sides, i.e., $\tilde{\phi}_{1,btm} = \tilde{\phi}_{1,top} + \alpha$ where α is the (unknown) constant scalar that will be solved for together with the unknown $\tilde{\boldsymbol{\Phi}}_1$ in the modified least-square problem described as follows.

For the convenience of implementation, we treat the free DOF (denoted as $\bar{\boldsymbol{\Phi}}_1$) and total DOF $\tilde{\boldsymbol{\Phi}}_1$ as two vectors. They are equal on all nodes excluding the bottom side of the gap where $\tilde{\phi}_{1,btm} = \bar{\phi}_{1,top} + \alpha$. The matrix representation of the linear relation between $[\bar{\boldsymbol{\Phi}}_1, \alpha]$ and $\tilde{\boldsymbol{\Phi}}_1$ is

$$\tilde{\boldsymbol{\Phi}}_1 = \boldsymbol{A} \begin{pmatrix} \bar{\boldsymbol{\Phi}}_1 \\ \alpha \end{pmatrix} \tag{19}$$

where $\boldsymbol{A}$ is a constant and sparse mapping matrix. With this notation, the modified least square problem is written as $\min_{\bar{\boldsymbol{\Phi}}_1, \alpha} \tilde{L}(\tilde{\boldsymbol{\Phi}}_1(\bar{\boldsymbol{\Phi}}_1, \alpha))$ with $\tilde{L}$ given in (16). Denote $\boldsymbol{G}$ and $\boldsymbol{H}$ as the global gradient and Hessian assembled from (17) and (18), respectively, the global gradient and Hessian for the modified least-square problem can be obtained by chain rule as $\bar{\boldsymbol{G}} = \boldsymbol{A}^{\mathrm{T}}\boldsymbol{G}$ and $\bar{\boldsymbol{H}} = \boldsymbol{A}^{\mathrm{T}}\boldsymbol{H}\boldsymbol{A}$, respectively, which are input to Newton's method.

The result of solving ϕ_i with the above treatment is demonstrated in Figures 4B and C which shows the ϕ_1 distribution of the flower-shaped cloak. It can be seen that ϕ_1 values on the two sides of the gap are different, but their gradients along the gap are identical. The graph (or surface) of ϕ_1 is similar to a helicoid, which is impossible for a non-simply connected domain.

Finally, to ensure structural smoothness at the gap, the feature size parameter needs to be chosen such that $\varepsilon = \frac{|\alpha|}{k}$ for some integer k. This ensures the ϕ_i on the two sides of the gap differ by an integer multiple of 2π and hence produces identical wave level set function values thereon.

5.3 Process of generating metamaterial structures

After obtaining the local microstructural parameters w_1 and w_2 (in 3.3) and the mapping ϕ_1 and ϕ_2 (in 5.1 and 5.2) as four scalar fields on the meta-device domain, we can generate the corresponding global structure through a wave level set function representation [5]. Specifically, we need two level set functions to represent the two groups of orthogonal laminates in the global structure, respectively. The level set functions are

$$\rho_i(r,\theta) = -\cos\left(\frac{2\pi}{\varepsilon_i}\phi_i(r,\theta)\right) + \cos\left(\pi w_i(r,\theta)\right),\ i = 1,2 \tag{20}$$

where the feature size parameter ε_i is user-defined and needs to satisfy the mild requirement discussed in 5.2.

To illustrate the procedure, we use the cloak as an example as shown in Supplementary Figure 5. Given the four (discrete) fields of w_1, w_2, ϕ_1, and ϕ_2, we obtain the level set functions ρ_1 and

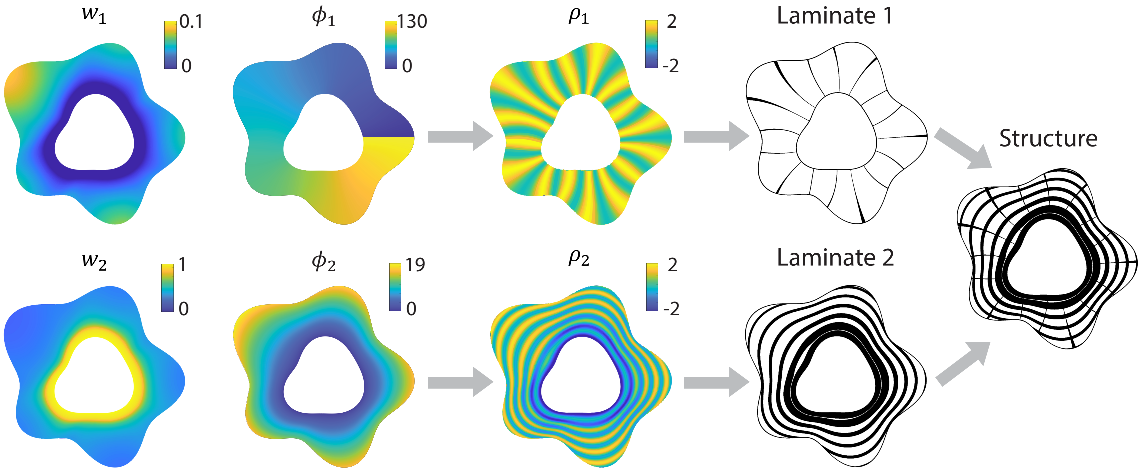

Supplementary Figure 5: Process to generate the meta-device (cloak) after obtaining w_1, w_2, ϕ_1, and ϕ_2.

ρ_2 using (20) with values plotted in the third column of Supplementary Figure 5. Based on [5], the Material A (steel) parts for the first and second laminates are defined by the regions $\rho_1 \leq 0$ and $\rho_2 \leq 0$, respectively, which are shown in the fourth column of Supplementary Figure 5. Note that relative widths and orientations of the laminates are consistent with the distributions of w_i and ϕ_i by construction. Finally, the metamaterial structure is obtained as the union of the two laminates as shown in the right-most plot of Supplementary Figure 5, and the union set can be expressed as $\rho_{min} \leq 0$ with $\rho_{min} := \min\{\rho_1, \rho_2\}$. We want to emphasize that the process of generating the structure, i.e., computing the level set functions and taking the union, requires negligible computational cost and is free of post-processing for structural connectivity, which is a major advantage over state-of-the-art, computation-based approaches reviewed in the introduction.

6 Supplementary Note 6: Numerical simulation of heat conduction

After obtaining the realization of the meta-device, we post-evaluate its heat conduction response using FEM. The steady-state 2D heat conduction problem is:

$$\begin{aligned} \nabla \cdot (\boldsymbol{\kappa} \nabla T) &= 0 && \text{in } \Omega \\ T &= \bar{T}_H && \text{on } \partial\Omega_{T,H} \\ T &= \bar{T}_L && \text{on } \partial\Omega_{T,L} \\ -\boldsymbol{\kappa} \nabla T &= 0 && \text{on } \partial\Omega_q \end{aligned} \tag{21}$$

where Ω is the domain, $\partial\Omega_{T,H}$ and $\partial\Omega_{T,L}$ are the Dirichlet boundaries of the hot and cold ends, respectively, and $\partial\Omega_q$ are the adiabatic Neumann boundaries, such that $(\partial\Omega_{T,H} \cup \partial\Omega_{T,L}) \cup \partial\Omega_q = \partial\Omega$ and $(\partial\Omega_{T,H} \cup \partial\Omega_{T,L}) \cap \partial\Omega_q = \varnothing$, $\bar{T}_H$ and $\bar{T}_L$ are the prescribed temperatures for the hot and cold ends, respectively. Note that we focus on the case with no internal heat sources or sinks.

The boundary value problem (21) is solved using an in-house FEM program where the four-node quadrilateral element with four Gauss points is adopted. The $\boldsymbol{\kappa}$ is assumed to be piece-wise constant and associated with the elements. Specifically, $\boldsymbol{\kappa} = \kappa_{\text{Steel}} \boldsymbol{I}$ for the steel parts of the structure, $\boldsymbol{\kappa} = \kappa_{\text{PDMS}} \boldsymbol{I}$ for the PDMS parts, and $\boldsymbol{\kappa} = \kappa_0 \boldsymbol{I}$ for the background. The square-shaped domain is discretized by a regular 2000×2000 mesh to allow for sufficient resolution of the microstructure.

7 Supplementary Note 7: Performance measures

This subsection presents the quantitative performance measures for the thermal meta-devices. The literature uses various more or less meaningful approaches to measure the meta-devices' performance.

Here, we adopt measures that output 0% when the performance is ideal and 100% when the meta-device is absent, i.e., with a homogeneous background (for the concentrator, the performance measure at the homogeneous medium case is not 100%). This represents a fair and transparent performance evaluation.

For the thermal cloak, we use the Relative Temperature Difference (RTD) as the performance measure which characterizes the averaged difference in the background temperature between the cloak and reference (homogeneous) cases relative to the bare case (with object and without cloak). It is defined as [7]

$$\mathrm{RTD} = \frac{\sum_{\Omega_\mathrm{b}} \left(T_\mathrm{cloak} - T_\mathrm{ref}\right)^2}{\sum_{\Omega_\mathrm{b}} \left(T_\mathrm{bare} - T_\mathrm{ref}\right)^2} \tag{22}$$

where Ω_b denotes the background region outside the cloak. The RTD value takes 1 when there is no cloak, and 0 when there is an ideal cloak.

For the thermal rotator, we use the Relative Angular Difference (RAD) as the performance measure. RAD measures the average difference between the core's flux angle and the prescribed rotation angle relative to the prescribed angle. It is defined as

$$\mathrm{RAD} = \frac{\sum_{\Omega_\mathrm{c}} \left(\rho_\mathrm{rotator} - \rho^*\right)^2}{N_c \rho^{*2}} \tag{23}$$

where Ω_c is the core region, ρ^* is the prescribed rotation angle, and N_c is the number of elements in the core region. The RAD value takes 1 when there is no heat rotation, and takes 0 when the rotated heat in the core is perfectly aligned in the prescribed direction.

For the thermal concentrator, we use the Relative Gradient Difference (RGD) to measure the average difference between the core's flux magnitude and the prescribed rate of concentration relative to the prescribed rate. It is defined as

$$\mathrm{RGD} = \frac{\sum_{\Omega_\mathrm{c}} \left(\|\nabla T\|_\mathrm{concentrator} - \|\nabla T\|^*\right)^2}{N_c \|\nabla T\|^{*2}} \tag{24}$$

where $\|\nabla T\|^* := c\frac{T_1 - T_0}{h}$ with T_1 and T_0 being the constant temperatures applied at the background's opposite boundaries, h being the dimension of the background in the applied heat direction, and c being the specified rate of concentration.

8 Supplementary Note 8: Extended investigations

8.1 SI: Cloak with different core materials

An ideal thermal cloak should hide any objects at the core from the external temperature field. Here, we numerically verify this phenomenon using the de-homogenized cloaks. We test four different core materials (air, PDMS, steel, and aluminum) covering a wide range of thermal conductivity. The temperature fields of the cloaked and bare cases under vertically applied heat are shown in Supplementary Figure 6 together with the RTD values. It can be seen that the RTD values of the cloaked cases are small for all core materials and insensitive to the change of conductivity. By contrast, the core material's conductivity has a significant impact on the temperature distribution of the bare case.

8.2 SI: Influence of feature size on rotator and concentrator

We investigate the influence of feature size on the performance of the rotator and concentrator. The rotator and concentrator with four different relative feature sizes are shown in Figures 7 A and B, respectively, with size 1 investigated (and tested) in the main text. Generally, an increase in feature size worsens the performance but to different levels. The rotator's performance is more sensitive to feature size as the RAE value increases rapidly as the size grows. By contrast, the concentrator is less sensitive in that the RGE value increases more slowly. These observations, however, are case-dependent and may not be general.

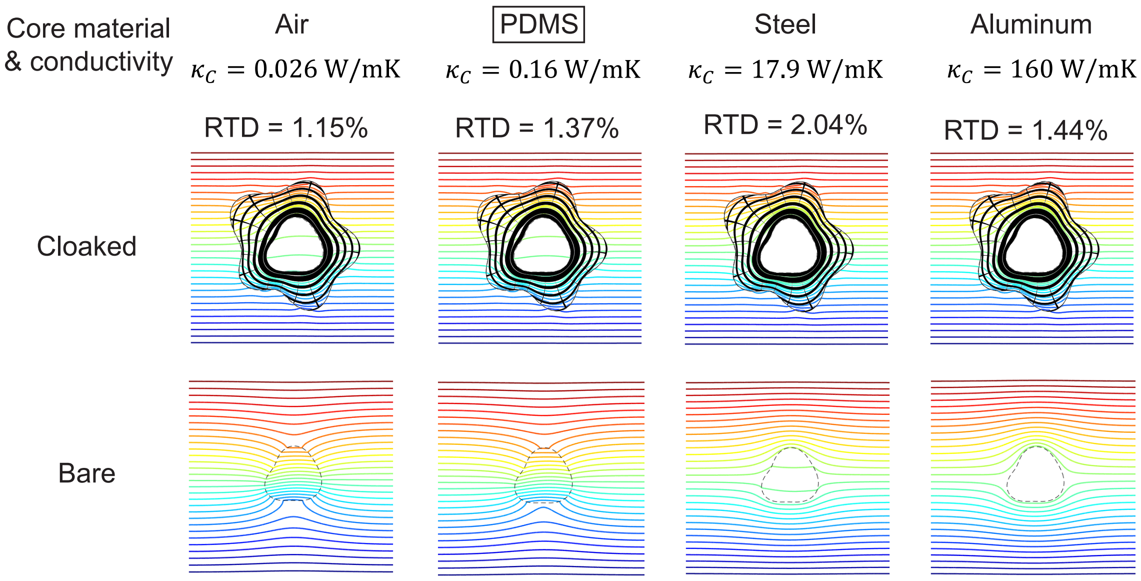

Supplementary Figure 6: Influence of core material on the cloaking effect.

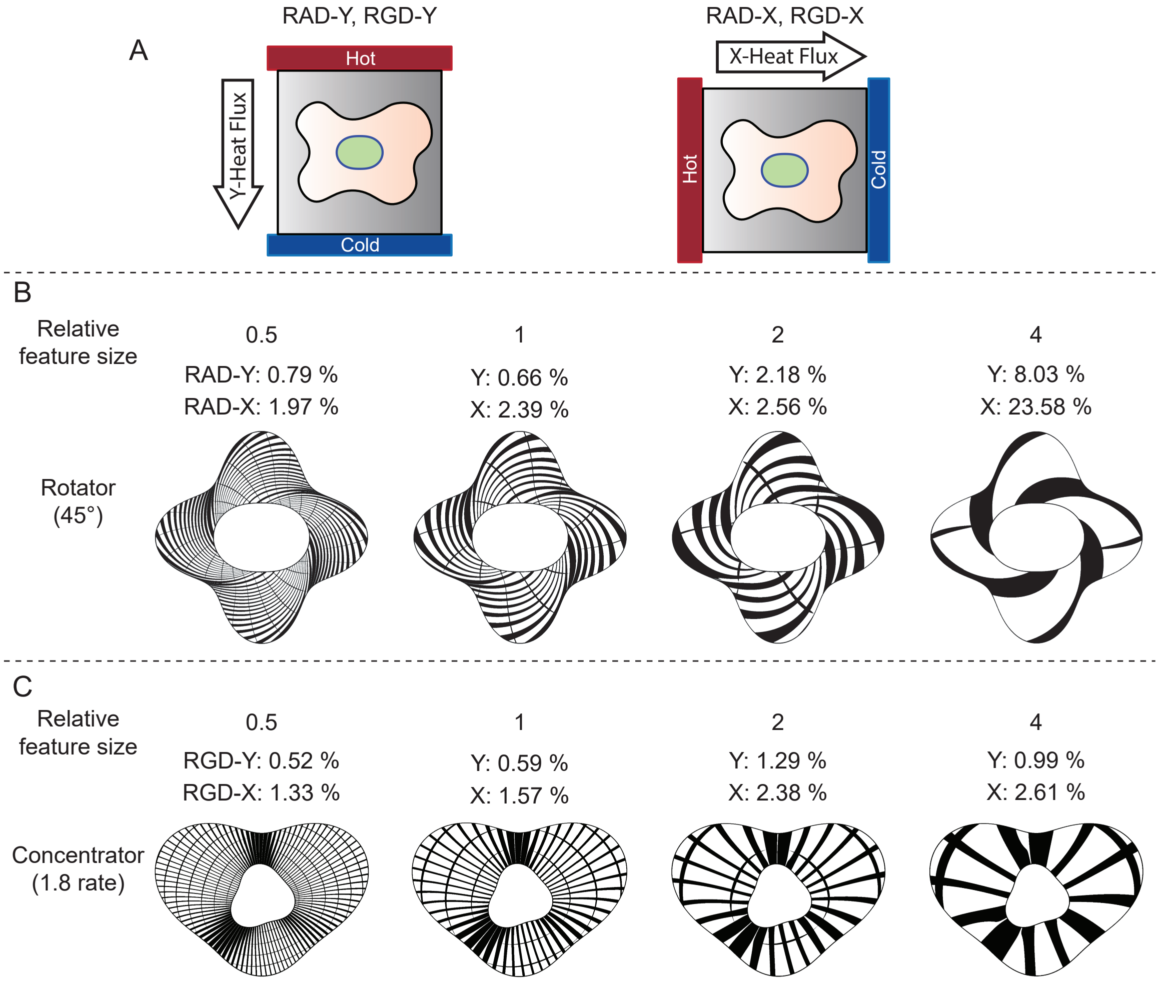

Supplementary Figure 7: Influence of feature size on the metastructure and performance. A) Illustration of Y-direction and X-direction applied heat and corresponding performance measure; B) & C) feature size's influence on rotator and concentrator, respectively.

9 Supplementary Note 9: Fabrication and experimental setup

This section documents the fabrication details of the thermal meta-device. The 3D-printed metal part is placed into a 3D-printed PLA mold pre-filled with liquid PDMS. The mold containing the metal part and PDMS is degassed under -0.8 atmospheric pressure for one hour to remove bubbles. Then, they are placed into an oven and cured at 60 C° for 4 hours, after which the PLA mold is removed, and the spurious PDMS on the side surfaces of the metal parts are also removed with a utility knife. The metal+PDMS part is then placed in the middle of another square-shaped PLA mold for casting the encapsulant into the background. After casting, the setup is degassed for one hour to remove the bubbles in the background. The whole setup is placed in the oven at 60 C° for four hours to cure the background. Finally, the PLA mold is removed with the utility knife.

For the experiment, the top end of the square-shaped material is inserted into an electric heating metal plate with a slot and the bottom end is inserted into another metal plate soaked in a tank of iced water. Thermal grease is applied at the slots to ensure a smooth heat path. To better represent the adiabatic boundary condition on the left and right ends, we clamp two foam bars onto the two sides. The heating plate's temperature is kept constant at 37 C° through a heating controller. After the material is installed, we wait for 45 minutes for the material to reach the steady state after which the temperature profiles are reported.

References

[1] Allan Roulund Gersborg and Ole Sigmund. Extreme non-linear elasticity and transformation optics. *Opt. Express*, 18(18):19020–19031, Aug 2010.

[2] Ji-Ping Huang. *Theoretical Thermotics: Transformation Thermotics and Extended Theories for Thermal Metamaterials*. Springer, 2020.

[3] Jeroen P. Groen and Ole Sigmund. Homogenization-based topology optimization for high-resolution manufacturable microstructures. *International Journal for Numerical Methods in Engineering*, 113(8):1148–1163.

[4] Jeroen P. Groen, Florian C. Stutz, Niels Aage, Jakob A. Bærentzen, and Ole Sigmund. Dehomogenization of optimal multi-scale 3d topologies. *Computer Methods in Applied Mechanics and Engineering*, 364:112979, 2020.

[5] Grégoire Allaire, Perle Geoffroy-Donders, and Olivier Pantz. Topology optimization of modulated and oriented periodic microstructures by the homogenization method. *Computers Mathematics with Applications*, 78(7):2197–2229, 2019.

[6] The MathWorks Inc. Matlab version: 9.9.0.1467703 (r2020b), 2020.

[7] Yihui Wang, Wei Sha, Mi Xiao, Cheng-Wei Qiu, and Liang Gao. Deep-learning-enabled intelligent design of thermal metamaterials. *Advanced Materials*, 35(33):2302387, 2023.
