# Supplementary material for: Analytical realization of complex thermal meta-devices
Source: Nat Commun. 2024 Jul 15;15:5527. doi: 10.1038/s41467-024-49630-1 (PMC11250795; doi:10.1038/s41467-024-49630-1)
Supplement: Supplementary file 3 — Description of Additional Supplementary Files [file 41467_2024_49630_MOESM3_ESM.pdf]

Description of Additional Supplementary Files

File Name: Supplementary Movie 1

Description: Experiment of thermal cloak

File Name: Supplementary Movie 2

Description: Experiment of thermal rotator

File Name: Supplementary Movie 3

Description: Experiment of thermal concentrator
